# Analyzing Interaction of μ-, δ- and κ-opioid Receptor Gene Variants on Alcohol or Drug Dependence Using a Pattern Discovery-based Method

**Published:** 2013-05-14

**Authors:** Zhong Li, Huiping Zhang

**Affiliations:** 1 Department of Computational Genetics, High Throughput Biology Inc., Summit, NJ, USA; 2 Department of Psychiatry, Yale University School of Medicine, New Haven, CT, USA; 3 VA Connecticut Healthcare System, West Haven Campus, CT, USA

**Keywords:** Opioid receptor genes, Case-control genetic association study, Gene-gene interaction, Pattern discovery-based association test

## Abstract

**Background:**

Polymorphisms in the μ-, δ- and κ-opioid receptor genes (*OPRM1*, *OPRD1* and *OPRK1*) have been reported to be associated with substance (alcohol or drug) dependence. The influence of an individual gene on a disease trait should be more evident when analyzed in the context of gene-gene interactions. Thus, we assessed the joint effect of variants in these three opioid receptor genes on alcohol, cocaine, or opioid dependence.

**Methods:**

Genotype data for 13 *OPRM1* Single Nucleotide Polymorphisms (SNPs), 11 *OPRD1* SNPs and seven *OPRK1* SNPs were obtained from 382 European Americans (EAs) affected with substance dependence [among them, 318 with Alcohol Dependence (AD), 171 with Cocaine Dependence (CD), and 91 with Opioid Dependence (OD)] and 338 EA control subjects. We assessed the joint effect of *OPRM1*, *OPRD1* and *OPRK1* variants on AD, CD, or OD using a pattern discovery-based association test. Specific marker patterns (consisting of alleles of *OPRM1*, *OPRD1* and *OPRK1*) that were significantly more frequent in AD, CD, or OD cases than in controls were identified.

**Results:**

12 significant patterns in the AD dataset, four significant patterns in the CD dataset, and 18 significant patterns in the OD dataset were identified. Moreover, the significance of most marker patterns was due primarily to *OPRM1* variants and, to a lesser degree, *OPRD1* variants.

**Conclusion:**

Our findings suggest that variation in the above three opioid receptor genes can jointly influence the vulnerability of individuals to alcohol or drug dependence. Evidence provided by this study also supports previous biological findings that the interaction of the three opioid receptors can modulate the action of opioid and non-opioid drugs and alcohol.

## Introduction

Substance dependence, such as alcohol, cocaine, or opioid dependence, is a set of genetically complex disorders due to the effect of a number of different individual disease genes (heterogeneity) or a combination of different disease genes (polygeneity). In addition, environmental factors also have a strong influence on the development of substance dependence. Given the high rate of co-morbidity of alcohol, cocaine, and opioid dependence, and consistent with studies in genetic epidemiology, it is likely that, besides specific genetic factors that are responsible for each of the substances abused, common genetic factors may be involved in these disorders as well [1,2]. There is evidence that the three opioid receptor genes (*OPRM1*, at 6q24-q25, which encodes the μ-opioid receptor; *OPRD1*, at 1p36.1-p34.3, which encodes the δ-opioid receptor; and *OPRK1*, at 8q11.2, which encodes the κ-opioid receptor) could be such common genetic factors [3–6].

The above three opioid receptors are the molecular targets for endogenous opioid peptides, opioid analgesic agents, and commonly abused opioid drugs like heroin. There is mounting evidence that the three receptors directly mediate reward, tolerance, and dependence associated with opioids [7,8]. They are also indirectly involved in the reinforcing properties of non-opioid drugs (such as cocaine and alcohol) due to the intimate relationship between the opioid system and the mesolimbic dopamine system. Dopamine is known to be a key neurotransmitter interacting with the brain reward center [9,10]. Cocaine binds to the dopamine transporter and inhibits dopamine re-uptake in the Nucleus Accumbens (NAc), thus increasing synaptic dopamine levels and stimulating dopaminergic transmission [11,12], whereas ethanol directly stimulates dopaminergic neurons in the Ventral Tegmental Area (VTA), leading to increased release of dopamine in the NAc [13]. The basal dopamine level in the dopamine system is under the tonic control of two opposing opioid systems: activation of the μ-receptor (and possibly the δ-receptor) in the VTA increases extracellular dopamine levels in the NAc; activation of the κ-receptor in the VTA decreases extracellular dopamine levels in the NAc [14,15].

Additionally, interaction of the three opioid receptors can modulate the action of opioid and non-opioid drugs and alcohol. There is evidence of physical and functional interactions between μ- and δ-opioid receptors. Extensive co-localization of μ- and δ-receptors has been observed in brain reward regions [16–18]. The apposition of μ- and δ-receptors suggests that these two receptors are functionally inter-related. Several studies have demonstrated modulatory interactions between μ- and -receptors. For example, δ-agonists can enhance the analgesic potency and efficacy of μ agonists (e.g., morphine), and δ-antagonists can prevent or diminish the development of tolerance and physical dependence by μ agonists [19,20]. Of interest, μ-δ heterodimers, which exhibit ligand binding and signalling characteristics distinct from those of μ- and δ-receptors, have been isolated from cells co-expressing these two receptors [21]. In contrast to the interaction between μ- and δ-receptors, opposing interactions have been observed between μ- and κ-receptors. Activation of the κ-receptor by κ-receptor agonists opposed a variety of μ-receptor mediated actions in the brain, including analgesia, tolerance, reward, and memory processes [22]. However, the inhibitory effect of κ-receptor agonists on the function of the μ-receptor can be completely reversed by the κ-receptor antagonist nor-BNI [23]. Similarly, opposing interactions have been observed between δ- and κ-opioid receptors. In addition, δ- and κ-receptors can form heterodimers that exhibit ligand binding and functional properties that are different from those of either receptor. The δ-κ heterodimer can bind highly selective agonists and potentiate signal transduction [24].

Considering the close biological interaction of the three receptors, we hypothesized that variation in their genes (*OPRM1*, *OPRD1,* and *OPRK1*) might have joint effects on risk for alcohol or drug dependence. We sought to test this hypothesis via a powerful multi-locus analysis method based on an efficient pattern discovery algorithm.

## Materials and Methods

### Sample and genotype data

As described in our two previous studies [4,6], genotype data of 13 *OPRM1* SNPs, 11 *OPRD1* SNPs, and seven *OPRK1* SNPs were obtained from 376 European American cases (280 males and 96 females), who met lifetime DSM-III-R (American Psychiatric Association, 1987) or DSM-IV (American Psychiatric Association, 1994) criteria for the diagnosis of alcohol, cocaine, or opioid dependence (AD, CD, or OD), and 384 European American healthy controls (143 males and 241 females). Among 376 cases, 318 were affected with AD, 166 were affected with CD, and 91 were affected with OD, respectively. The study subjects were recruited at the University of Connecticut Health Center or the VA Connecticut Healthcare System-West Haven Campus. The study protocol was approved by the Institutional Review Board (IRB) at each clinical site. Informed consent was obtained from participants before they entered the study, and they were paid for their participation.

### Multi-locus interaction and disease association analyses

The Pattern Examiner program [25,26], which is a pattern discovery-based association analysis approach, was applied in analyzing the interactive effect of variation in three opioid receptor genes (*OPRM1*, *OPRD1*, and *OPRK1*) on alcohol or drug dependence. Pattern Examiner is a non-parametric data mining method for detection of multi-locus gene-gene or gene-environment interactions in population-based case-control studies. This method has two major steps: (1) pattern discovery, and (2) significance evaluation. Briefly, data are organized in a two-dimensional matrix with markers as columns, individuals as rows, and individuals’ alleles or genotypes as cell values. Each marker is represented by five columns: two for each of the two alleles and three for each of the three possible genotypes. A pattern is defined as a maximal sub-matrix of the data matrix in which the value of each marker across all individuals in the sub-matrix satisfies a predefined equivalence criterion such as the same genotype value. A sub-matrix is maximal if (1) no more rows can be added while keeping the columns fixed, and (2) no more columns can be added while keeping the rows fixed. Under this formulation, patterns can be used to model both multi-locus allelic and multi-locus genotypic contributions to a disease state. In the pattern discovery step, patterns are identified using input data from the case population alone to uncover elevated risk factors enriched in the case population (patterns from the control population alone can also be examined to uncover protective factors). The extensiveness and execution time of the pattern discovery step are controlled by two parameters: the *support threshold,* which specifies the minimum number of rows a pattern must have; and the *locus threshold,* which specifies the extent of locus interaction. For example, with the support and locus thresholds set to 20 and 2, respectively, all reported patterns will have 20 or more case supports and mostly one or two markers. In the significance evaluation step, a 2×2 contingency table is constructed for each pattern to tally its support in the case and control populations (“case support” and “control support,” respectively). The two categorical variables tabulated are Population Type (“cases” vs. “controls”) and Pattern Match Status (“matches” vs. “does not match”). Partially missing data are excluded. *p* values are obtained from a ϕ^2^ test of independence and then adjusted for multiple testing.

To examine the interaction of *OPRM1*, *OPRD1*, and *OPRK1*, we performed a two-locus marker-based gene-gene interaction analysis using the above Pattern Examiner algorithm. The support threshold and the locus threshold parameters were set to 1 and 2, respectively, meaning that all reported patterns had at least one case support and no more than two loci were included in the analysis. The support threshold of 1 was chosen so that all possible patterns were identified and evaluated. A modified Bonferroni correction for multiple testing was applied to generate the adjusted *p* values for the identified patterns. The correction factor for the modified Bonferroni correction was the total number of patterns that contained equal or greater case support than the target pattern rather than the total number of patterns identified. As a result, the modified Bonferroni correction was less stringent than the direct Bonferroni correction (in which the correction factor was the total number of patterns identified) and thus yielded fewer false negatives. In both multiple testing correction schemes, the adjusted significance was robust against the arbitrary selection of values for the support threshold parameter in the pattern discovery step. The odds ratio with its confidence interval was also calculated for each pattern.

## Results

In our previous studies, we examined the association of 13 *OPRM1* SNPs, 11 *OPRD1*, and seven *OPRK1* SNPs (marker information is given in Table 1) with alcohol or drug dependence. Single marker and haplotype analyses have shown a positive association of variation in the three opioid receptor genes and alcohol or drug dependence [4,6]. In the present study, we further analyzed the interactive effect of *OPRM1*, *OPRD1*, and *OPRK1* variants on alcohol or drug dependence using a pattern discovery-based method.

**Figure 1:**
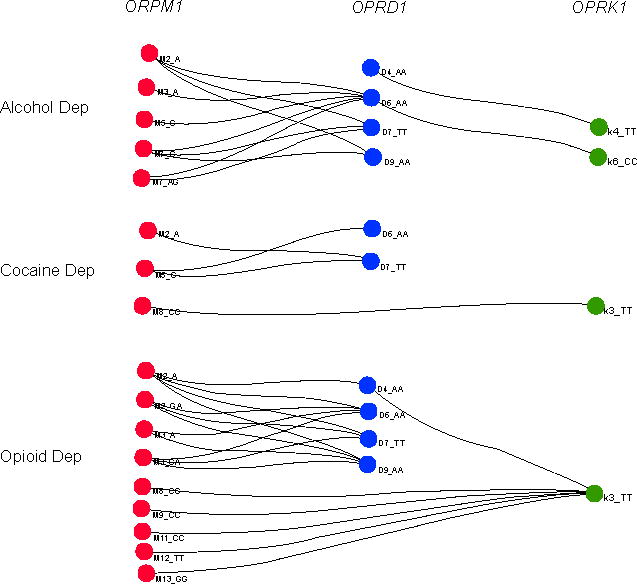
Interactive effects of *OPRM1*, *OPRD1*, and *OPRK1* variants on alcohol, cocaine, or opioid dependence. Two-locus marker-based gene-gene interaction analysis was performed using the program Pattern Examiner to indentify marker patterns which were significantly more frequent in cases than in controls. 12 significant patterns in the alcohol dependence (AD) dataset, four significant patterns in the cocaine dependence (CD) dataset, and 18 significant patterns in the opioid dependence (OD) dataset were identified.

Two-locus marker-based gene-gene interaction analysis results are presented in table 2. A small proportion of marker patterns [1.06% (12 of 1,134), 0.35% (4 of 1,147), and 1.86% (18 of 965) for alcohol, cocaine and opioid datasets, respectively] were found significantly more frequent in cases than in controls (*p*<0.05, after the adjusted Bonferroni correction). Figure 1 illustrates significant interactions among markers of *OPRM1, OPRD1*, and *OPRK1*. Each node in the graph represented a marker with a particular allele or genotype that was found in significant patterns. Each edge represented a significant pattern (*p* values were labelled on edges). The majority of the significant patterns were comprised of marker alleles of *OPRM1* and *OPRD1*, suggesting a greater impact of these two genes on alcohol, cocaine, or opioid dependence in comparison to that of *OPRK1*. Remarkably, one *OPRM1* SNP (M2) and two *OPRD1* SNPs (D6 and D7) were consistently present in significant patterns for all three substance dependence datasets, suggesting the existence of common disease variants or a combination of common disease variants for alcohol, cocaine, and opioid dependence. Moreover, several significant marker patterns appeared in two or all three datasets. The interaction of M2_A and D7_TT was noticed in all three datasets. Additionally, patterns M2_A~D6_AA, M2_A~D9_AA, and M3_A~D6_AA were shown in both alcohol and opioid dependence datasets, pattern M5_C~D6_AA was observed in both alcohol and cocaine dependence datasets, and pattern M8_CC~K3_TT was found in both cocaine and opioid dependence datasets. Interestingly, all markers of *OPRD1* and *OPRK1* in significant patterns contained homozygous genotypes, suggesting a recessive effect by *OPRD1* or *OPRK1* towards the disease etiology of alcohol, cocaine, or opioid dependence. A lesser consistent effect was observed for markers of *OPRM1*.

## Discussion

To our knowledge, this is the first study to look at the joint effect of the three opioid receptor genes on three complex substance dependence traits (alcohol, cocaine, and opioid dependence) that co-occur frequently. The gene-gene interaction results support the findings in our previous single gene studies [4,6]. The significance of most marker patterns (as presented in table 2 and figure 1) was due primarily to *OPRM1* markers and, to a lesser degree, *OPRD1* markers. A plausible explanation for this finding is that, among the three receptor genes, *OPRM1* variants produce the strongest effect on substance dependence; *OPRD1* variants combine with *OPRM1* variants to generate an additive (or possibly synergistic) effect; and *OPRK1* variants can modulate the effects of *OPRM1* or *OPRD1* variants. The weaker role of *OPRK1* variants in substance dependence observed in this study agrees with the findings in previous neuropsychopharmacological studies that the κ-opioid receptor seemed to mediate psychotomimetic effects [27], which do not have a clear relation to risk of substance dependence. In contrast, the μ-receptor (coded by *OPRM1*), in particular, and the δ-receptor (coded by *OPRD1*) to a lesser degree, play a major role in opioid drug reward and addiction.

In comparison to conventional single gene/marker association analysis, multi-locus association analysis may be more powerful. Single gene/marker analysis has been widely used to study many complex disorders. However, the findings are often inconsistent. The inconsistency may be due to insufficient sample size, population stratification, random variation, and confounding factors. The multi-factorial nature of complex disorders may also lead to inconsistent results. The interaction of several genes involved in a disease may complicate the findings. Although synergistic effects of multiple genes can be expected to augment the phenotypic expression of a disorder, in certain circumstances, the effect of one gene may be suppressed or opposed by another gene, and as a result, the influence of one gene in a disease may be rendered undetectable. In view of this, if the information concerning the interactive effect of genes is considered, the chance to detect the risk effect of a susceptibility locus will be increased, even when the sample size is moderate [28]. One more advantage of the pattern discovery-based association test is that, since both alleles and genotypes were included in the pattern search, it had the potential to reveal the mode of inheritance at each locus. In the two-locus marker-based analysis, a dominant or recessive mode of inheritance was seen for almost all significant patterns (Figure 1). For example, several markers of *OPRM1* in significant patterns showed a dominant effect as indicated by the inclusion of a single allele. On the contrary, several other markers of *OPRM1* and all markers of *OPRD1* and *OPRK1* consistently showed a recessive effect as indicated by the inclusion of homozygous genotypes in the significant patterns.

There are two major challenges to the use of multi-locus association analysis. One challenge is the combinatorial nature of gene-gene interaction analyses. Increasing the number of loci results in exponential growth of possible multi-locus combinations, thus leading to strenuous computation. The pattern discovery approach has been proved to be an efficient way to deal with the combinatorial nature of gene-gene interaction analyses [25]. Another challenge is how to adjust the statistical significance for multiple testing. Multi-locus combinations and strong correlations among different marker combinations due to locus sharing make the use of Bonferroni correction inappropriate. Here, we employed a modified Bonferroni correction scheme, in which the raw *P* value for a specific pattern was adjusted by the total number of patterns that have the same or more case support than this pattern instead of using all patterns identified (many of which may have lesser case support than this pattern). The validity of this multiple testing correction method was confirmed by Li et al. [25] using a Monte Carlo process [29,30].

In summary, the present study, by using a pattern discovery-based association test approach, has demonstrated a potential interactive effect of the three opioid receptor genes on substance dependence. Our data have shown the importance of assessing joint effects of multiple related genes on the susceptibility to complex disorders such as substance dependence. The disease association patterns identified in this study may be useful for diagnosis and prediction of substance dependence. Furthermore, these findings have important pharmacogenetic implications relevant to the treatment of substance dependence, which we would argue that the joint effect of the three opioid receptor genes must be taken into consideration.

## Figures and Tables

**Table 1:** Information and genotyping methods for *OPRM1*, *OPRD1*and *OPRK1* Single Nucleotide Polymorphisms (SNPs).

SNP ID (this study)	SNP ID (NCBI rs#)	Gene	Alleles	MAF^a^	Location	Amino Acid Change	Chromosome Position (bp)	Genotyping Method
M1	rs1799971	*OPRM1*	A118G	0.13 (G)	Exon 1	Asn40Asp	Chr06: 154402490	PCR-RFLP
M2	rs511435	*OPRM1*	A/G	0.21 (A)	Intron 1	-	Chr06: 154410240	TaqMan
M3	rs524731	*OPRM1*	A/C	0.20 (A)	Intron 1	-	Chr06: 154416785	TaqMan
M4	rs3823010	*OPRM1*	A/G	0.15 (A)	Intron 1	-	Chr06: 154420845	TaqMan
M5	rs495491	*OPRM1*	C/T	0.24 (C)	Intron 1	-	Chr06: 154424235	TaqMan
M6	rs1381376	*OPRM1*	A/G	0.15 (A)	Intron 1	-	Chr06: 154434951	TaqMan
M7	rs3778156	*OPRM1*	A/G	0.15 (G)	Intron 1	-	Chr06: 154446006	TaqMan
M8	rs2075572	*OPRM1*	C/G	0.43 (G)	Intron 2	-	Chr06: 154453697	TaqMan
M9	rs548646	*OPRM1*	C/T	0.34 (T)	Intron 3	-	Chr06: 154459840	TaqMan
M10	rs9322447	*OPRM1*	A/G	0.48 (A)	Intron 3	-	Chr06: 154466013	TaqMan
M11	rs609148	*OPRM1*	C/T	0.25 (T)	Intron 3	-	Chr06: 154472707	TaqMan
M12	rs648893	*OPRM1*	C/T	0.25 (C)	Intron 3	-	Chr06: 154480321	TaqMan
M13	rs671531	*OPRM1*	A/G	0.35 (A)	downstream		Chr06: 154482434	TaqMan
D1	rs569356	*OPRD1*	C/T	0.12 (C)	upstream		Chr01: 29009273	PCR-RFLP
D2	rs1042114	*OPRD1*	G80T	0.13 (G)	exon 1	Cys27Phe	Chr01: 29011562	PCR-RFLP
D3	rs678849	*OPRD1*	C/T	0.47 (C)	intron 1	-	Chr01: 29017775	PCR-RFLP
D4	rs2236857	*OPRD1*	A/G	0.30 (G)	intron 1	-	Chr01: 29034196	TaqMan
D5	rs2236855	*OPRD1*	G/T	0.29 (T)	intron 1	-	Chr01: 29034586	TaqMan
D6	rs2298896	*OPRD1*	A/C	0.37 (C)	intron 1	-	Chr01: 29038725	TaqMan
D7	rs421300	*OPRD1*	C/T	0.38 (C)	intron 1	-	Chr01: 29042180	TaqMan
D8	rs529520	*OPRD1*	G/T	0.50 (T)	intron 1	-	Chr01: 29047533	TaqMan
D9	rs12749204	*OPRD1*	A/G	0.21 (G)	intron 1	-	Chr01: 29048800	TaqMan
D10	rs2234918	*OPRD1*	C921T	0.44 (C)	exon 3	Gly307Gly	Chr01: 29062184	PCR-RFLP
D11	rs204076	*OPRD1*	A/T	0.34 (A)	downstream		Chr01: 29062977	TaqMan
K1	rs12675595	*OPRK1*	A/G	0.09 (A)	upstream		Chr08: 54330478	TaqMan
K2	rs1051660	*OPRK1*	G36T	0.10 (T)	exon 1	Pro12Pro	Chr08: 54326115	TaqMan
K3	rs6985606	*OPRK1*	C/T	0.46 (T)	intron 1	-	Chr08: 54323669	TaqMan
K4	rs997917	*OPRK1*	C/T	0.34 (C)	intron 1	-	Chr08: 54314931	TaqMan
K5	rs702764	OPRK1	C843T	0.12 (C)	exon 3	Ala281Ala	Chr08: 54304710	TaqMan
K6	rs963549	OPRK1	C/T	0.14 (T)	exon 3 (UTR)		Chr08: 54304377	PCR-RFLP
K7	rs7820807	OPRK1	C/T	0.12 (C)	downstream		Chr08: 54301414	TaqMan

a Marker minor allele frequency (MAF) in European American (EA) healthy control subjects

**Table 2:** Significant marker patterns identified in two-locus marker-based gene-gene interaction analyses.

Dataset	Num. of cases with/ without a pattern	Num. of controls with/ without a pattern	Unadjusted *P* value	Adjusted *P* value	Odds Ratio (Confidence Interval)	Marker patterns
Alcohol Dependence (12 patterns)	71/207	37/268	3.19×10^−5^	0.007	2.48 (1.60–3.85)	M5_C + D6_AA
	89/181	57/247	1.00×10^−4^	0.013	2.11 (1.44–3.11)	D4_AA + K4_TT
	62/215	31/276	5.13×10^−5^	0.014	2.57 (1.61–4.09)	M2_A + D6_AA
	60/218	29/277	4.87×10^−5^	0.014	2.63 (1.63–4.23)	M3_A + D6_AA
	109/163	75/215	3.00×10^−4^	0.024	1.92 (1.34–2.74)	D6_AA + K6_CC
	76/200	45/259	2.00×10^−4^	0.03	2.19 (1.45–3.30)	M7_G + D9_AA
	46/232	19/285	8.25×10^−5^	0.032	2.97 (1.70–5.21)	M7_AG + D6_AA
	60/216	31/276	1.00×10^−4^	0.034	2.47 (1.55–3.95)	M2_A + D7_TT
	87/188	57/250	3.00×10^−4^	0.034	2.03 (1.38–2.98)	M2_A + D9_AA
	52/225	24/280	1.00×10^−5^	0.038	2.70 (1.61–4.51)	M7_G + D7_TT
	52/226	24/280	1.00×10^−4^	0.04	2.68 (1.60–4.49)	M7_G + D6_AA
	45/232	19/285	1.00×10^−4^	0.049	2.91 (1.66–5.11)	M7_AG + D7_TT
Cocaine Dependence (4 patterns)	39/102	37/268	5.13×10^−5^	0.001	2.77 (1.67–4.59)	M5_C + D6_AA
	37/103	37/268	2.00×10^−4^	0.037	2.60 (1.56–4.33)	M5_C + D7_TT
	33/107	31/276	2.00×10^−4^	0.043	2.75 (1.60–4.71)	M2_A + D7_TT
	19/120	11/293	9.67×10^−5^	0.047	4.22 (1.95–9.13)	M8_CC + K3_TT
Opioid Dependence (18 patterns)	33/44	57/249	7.74×10^−6^	0.0004	3.28 (1.92–5.60)	M2_A + D9_AA
	20/58	24/282	1.06×10^−5^	0.002	4.05 (2.10–7.82)	M2_GA + D6_AA
	22/56	30/275	2.45×10^−5^	0.005	3.60 (1.94–6.70)	M11_CC + K3_TT
	22/55	31/275	2.87×10^−5^	0.005	3.55 (1.88–6.46)	M12_TT + K3_TT
	30/47	55/250	8.25×10^−5^	0.005	2.90 (1.69–4.99)	M3_A + D9_AA
	13/63	11/292	1.70×10^−5^	0.006	5.48 (2.35–12.79)	M8_CC + K3_TT
	22/56	31/275	3.74×10^−5^	0.007	3.49 (1.88–6.46)	M2_A + D6_AA
	27/50	47/259	9.17×10^−5^	0.01	2.98 (1.70–5.22)	M2_GA+ D9_AA
	19/59	24/281	3.94×10^−5^	0.01	3.77 (1.94–7.33)	M3_CA + D6_AA
	18/60	22/284	4.15×10^−5^	0.012	3.87 (1.96–7.66)	M13_GG + K3_TT
	19/59	25/281	6.33×10^−5^	0.016	3.62 (1.87–7.00)	M2_GA + D7_AA
	20/58	28/278	8.70×10^−5^	0.02	3.42 (1.81–6.49)	D4_AA + K3_TT
	18/60	23/282	7.83×10^−5^	0.022	3.68 (1.87–7.24)	M3_CA + D7_TT
	26/51	46/259	2.00×10^−4^	0.023	2.87 (1.63–5.06)	M3_CA+ D9_AA
	21/57	31/275	1.00×10^−4^	0.024	3.27 (1.75–6.09)	M2_A + D7_TT
	18/60	24/282	1.00×10^−4^	0.034	3.52 (1.80–6.90)	M9_CC + K3_TT
	20/58	29/276	1.00×10^−4^	0.035	3.28 (1.74–6.20)	M3_A + D6_AA
	25/53	44/262	3.00×10^−4^	0.041	2.81 (1.58–4.98)	M2_A + D4_AA

Dataset: genotype data of controls and alcohol, cocaine, or opioid dependent cases.
